# Intensified and Accelerated Rescue Infliximab Therapy for Acute Severe Ulcerative Colitis in Pregnancy: A Case Report

**DOI:** 10.1002/jgh3.70091

**Published:** 2025-01-07

**Authors:** Saiumaeswar Yogakanthi, Timothy Phan, Emma Flanagan, Linda Yang, Chamara Basnayake, Iniyaval Thevathasan, Julien Schulberg

**Affiliations:** ^1^ Department of Gastroenterology St Vincent's Hospital Melbourne Fitzroy Victoria Australia; ^2^ Department of Medicine University of Melbourne Melbourne Victoria Australia

**Keywords:** clinical intestinal disorders, gastroenterology, immunology, microbiology and inflammatory bowel diseases

## Abstract

Acute severe ulcerative colitis (ASUC) in pregnancy poses a clinical challenge with significant risk to both mother and fetus. Anti‐TNF alpha therapy is known to be safe in pregnancy, however, data surrounding outcomes in ASUC is limited. In this report, we present the case of a pregnant patient of 10 weeks' gestation with ASUC successfully managed with intensified and accelerated infliximab therapy for a total dose of 35 mg/kg during a single admission. This case highlights the use of this therapeutic strategy, as a part of a multidisciplinary approach, to avoid the need for a high‐risk colectomy.

## 1 Introduction

Acute severe ulcerative colitis (ASUC) in pregnancy poses risks to both the mother and fetus. The condition represents a significant clinical challenge when initial therapy with intravenous steroids fails, secondary to a restricted therapeutic armamentarium in pregnancy, and a paucity of literature to guide decision making. Anti‐TNF alpha therapy with infliximab is known to be safe in pregnancy, however, limited data is available regarding outcomes with ASUC in this population [1]. Here, we present a case of a pregnant patient successfully managed with intensified and accelerated rescue infliximab therapy.

## 2 Case Report

A 29‐year‐old G1P0 10‐week gestation woman was transferred to our hospital from a regional center following presentation 1 day prior with up to 15 bloody bowel actions daily for 4 weeks. She was a non‐smoker and was otherwise well without comorbidity. On her index admission in 2019, she required IV hydrocortisone but no salvage drug therapy. She was on mesalamine 2.4 g BD and had previous treatment with azathioprine which was self‐ceased prior to pregnancy. Her fecal calprotectin on presentation was 2610 μg/g and colonoscopy the year prior showed moderate severity pancolitis.

On admission her CRP was 126 mg/L, albumin 24 g/L and hemoglobin 108 g/L. She was afebrile with normal vital signs. Physical examination demonstrated a soft abdomen with generalized tenderness. No toxic megacolon was present on abdominal x‐ray and stool cultures were negative. She was commenced on intravenous hydrocortisone 100 mg QID, rectal Budesonide, and prophylactic enoxaparin. Intravenous piperacillin‐tazobactam was also commenced due to initial concerns for infection. Flexible sigmoidoscopy on Day 3 showed severe colitis (Mayo score 3) to 25 cm. Histology was negative for CMV. 10 mg/kg of infliximab was administered on the same day. She was referred to the colorectal surgeons for review and consideration of a colectomy as well as the maternal‐fetal medicine obstetricians. With a BMI of 17.4 kg/m^2^, a dietician subjective assessment as moderate malnutrition, and the increased requirements of pregnancy, parental nutrition (PN) was commenced.

Improvement in CRP was noted following infliximab, however, due to ongoing elevation in bowel frequency a further 10 mg/kg dose was administered on Day 7 and Day 14. Intestinal ultrasound, performed on Day 14, showed moderate–severe inflammation from the sigmoid to proximal transverse colon [2]. A repeat flexible sigmoidoscopy on Day 15 demonstrated moderate colitis (Mayo score 2) disease in the rectum and severe colitis (Mayo score 3) between 20 and 40 cm. Fetal ultrasound showed a viable and healthy‐looking fetus.

**FIGURE 1:**
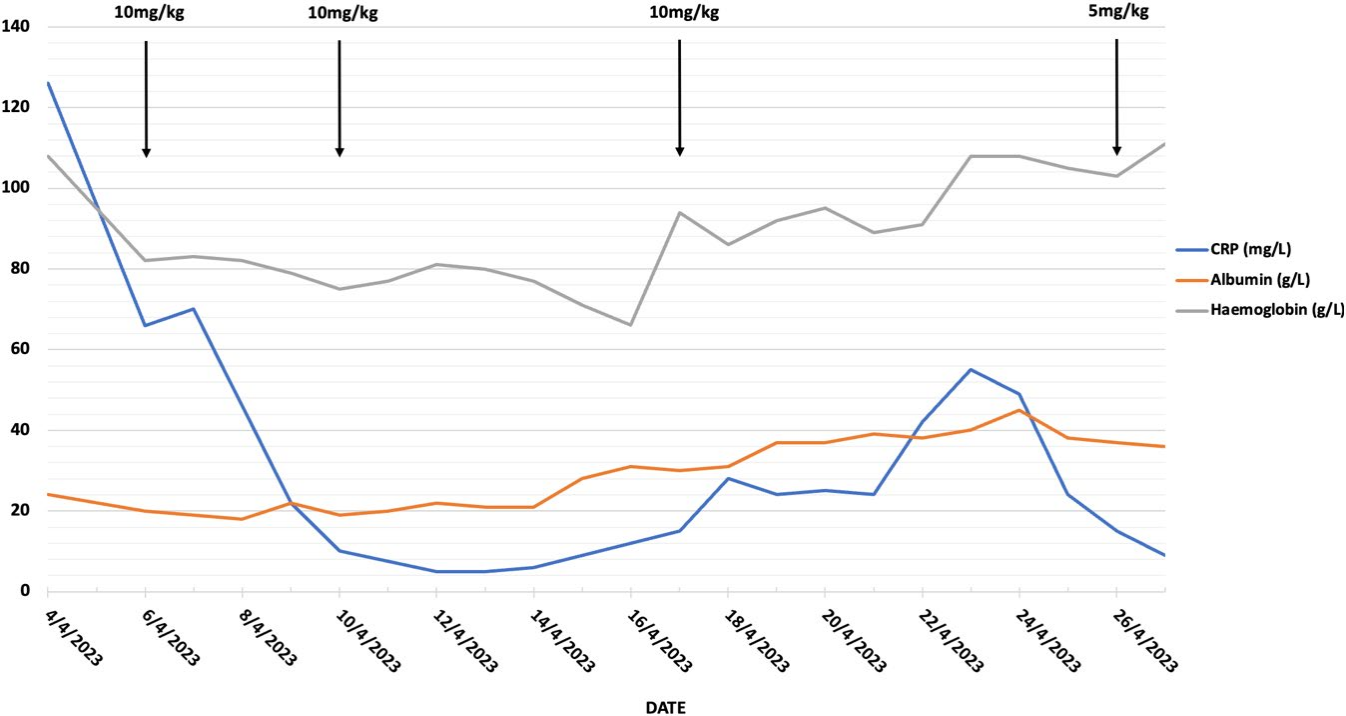
Trend of CRP, albumin and hemoglobin and infliximab dosing by date. Two units of packed red blood cells administered on the 16/4/23 and 2 × 20% concentrated albumin 100 mLs administered daily from the 14/4/23 to the 21/4/23.

Over the subsequent week, reducing bowel frequency and pain were noted. Febrile episodes on Day 18 and a rise in CRP despite no increase in bowel frequency prompted a septic screen consisting of a chest x‐ray, blood, urine, and stool cultures including clostridium difficile toxin and re‐commencement of antibiotics. The peripherally inserted central catheter, used for PN administration, was removed with subsequent defervescence, suggesting an infection may have been the cause. Following further clinical improvement with reduced bowel frequency and the absence of rectal bleeding, the patient's corticosteroids were tapered, and a further 5 mg/kg of infliximab was administered on Day 23 for a total of 35 mg/kg in 20 days. She was discharged 2 days later with a plan for a repeat infliximab dose in 4 weeks. She was 13 weeks' gestation at discharge and subsequently gave birth at 37 weeks to a healthy infant without further hospitalization (Figure 1).

## 3 Discussion

Our case highlights the challenges posed to treating clinicians by the occurrence of acute severe ulcerative colitis in pregnancy. Prospective data in ulcerative colitis comparing pregnant to non‐pregnant women has demonstrated that pregnancy is an established risk factor for disease flares with this risk greatest in the first and second trimesters [3]. Whilst data specific to ASUC is limited, the impact of inflammatory bowel disease (IBD) broadly upon pregnancy outcomes is well established. Meta‐analysis has demonstrated that women with IBD are more likely to have pre‐term delivery, stillbirth and small for gestational age birth weight newborns compared to healthy controls [4]. An increased risk of gestational diabetes regardless of corticosteroid use and preterm pre‐labor rupture of membranes have also been identified [5]. Literature regarding pregnancy outcomes in ASUC is restricted to case series and retrospective observational studies. The largest study of 20 patients by Ollech reported live births in 18 (90%) patients, with 6 (30%) premature births at a median gestational age of 33 weeks, 4 (20%) low birth weight infants, and no stillbirths [6]. Active disease at conception significantly increases the risk of consequent active disease during pregnancy, emphasizing the importance of achieving remission prior to planned pregnancy [7].

In patients with acute severe ulcerative colitis in pregnancy, the therapeutic management is concordant with non‐pregnant patients, consisting of intravenous steroids followed by rescue therapy in non‐responders to avoid colectomy. Whilst corticosteroid use in pregnancy is considered low risk overall, recommendations are made to utilize steroid‐sparing strategies to minimize the risk of potential fetal adverse effects such as pre‐term birth, low birth weight, intrauterine growth retardation, and neonatal intensive care unit admission [5]. Anti‐TNF alpha therapy with infliximab is demonstrated to be safe in pregnancy and hence was the preferred choice of rescue therapy in our case [1, 5]. Previous series by Ollech et al. found that 10 of 20 ASUC patients required anti‐TNF alpha rescue therapy with only one patient requiring a colectomy during index admission, although the infliximab dosing regimens were not documented [6]. Given only partial response to initial dosing in our case, sequential rescue therapy and colectomy were both considered. In the setting of limited safety data in pregnancy and concerns for teratogenicity in animal studies, the use of a JAK inhibitor was not chosen [5, 8]. Similarly, while cyclosporine was considered, the limited data surrounding its use in pregnancy for ASUC and concerns regarding risk of sequential rescue therapy made this option unfavorable, leaving colectomy as the main alternative [5, 9]. There is a scarcity of data surrounding the safety of surgery in pregnant patients with ulcerative colitis, though colectomy is suggested to be required in around 7.7% of ASUC cases during index admission in pooled analysis [6]. A systematic review examining pregnancies with complicated or medically refractory ulcerative colitis identified 56 cases, including some from more than 30 years ago. They reported a 19% maternal mortality and 50% pre‐term birth in the overall cohort, and a 35% fetal death rate in the subgroup with medically refractory ulcerative colitis [10]. The more recent SCAR study reported on 10 colectomies with 3 reported fetal losses (30%) but no maternal mortality [11]. Given the substantial risks of colectomy in our patient, an intensified and accelerated infliximab dosing strategy was used. To our knowledge, the cumulative infliximab dose of 35 mg/kg, administered over less than 3 weeks as an inpatient, is the largest cumulative dose reported in the literature for a pregnant ASUC patient.

In conclusion, acute severe ulcerative colitis (ASUC) in pregnancy poses significant management challenges. Failure to adequately manage these patients can have significant implications for both mother and the fetus. In the setting of failure to respond to initial therapy with intravenous steroids, further management options require careful consideration, including the potential for colectomy. Through utilization of a multidisciplinary team approach, this case illustrates the successful avoidance of a high‐risk colectomy in a pregnant patient with ASUC using intensified and accelerated rescue infliximab dosing, highlighting its use as a therapeutic strategy.

## Conflicts of Interest

The authors declare no conflicts of interest.
